# Focusing of Particles in a Microchannel with Laser Engraved Groove Arrays

**DOI:** 10.3390/bios11080263

**Published:** 2021-08-04

**Authors:** Tianlong Zhang, Yigang Shen, Ryota Kiya, Dian Anggraini, Tao Tang, Hanaka Uno, Kazunori Okano, Yo Tanaka, Yoichiroh Hosokawa, Ming Li, Yaxiaer Yalikun

**Affiliations:** 1 Division of Materials Science, Graduate School of Science and Technology, Nara Institute of Science and Technology, Ikoma 630-0192, Japan; zhang.tianlong.zo2@ms.naist.jp (T.Z.); kiya.ryota.kj8@ms.naist.jp (R.K.); dian.anggraini.cu7@ms.naist.jp (D.A.); tang.tao.ts3@ms.naist.jp (T.T.); uno.hanaka.uc7@ms.naist.jp (H.U.); k-okano@ms.naist.jp (K.O.); hosokawa@ms.naist.jp (Y.H.); 2 School of Engineering, Macquarie University, Sydney 2122, Australia; 3 Center for Biosystems Dynamics Research, RIKEN, Osaka 565-0871, Japan; yigang.shen@riken.jp (Y.S.); yo.tanaka@riken.jp (Y.T.)

**Keywords:** focusing, groove arrays, sensor applications, nanoparticle separation

## Abstract

Continuous microfluidic focusing of particles, both synthetic and biological, is significant for a wide range of applications in industry, biology and biomedicine. In this study, we demonstrate the focusing of particles in a microchannel embedded with glass grooves engraved by femtosecond pulse (fs) laser. Results showed that the laser-engraved microstructures were capable of directing polystyrene particles and mouse myoblast cells (C2C12) towards the center of the microchannel at low Reynolds numbers (Re < 1). Numerical simulation revealed that localized side-to-center secondary flows induced by grooves at the channel bottom play an essential role in particle lateral displacement. Additionally, the focusing performance proved to be dependent on the angle of grooves and the middle open space between the grooves based on both experiments and simulation. Particle sedimentation rate was found to critically influence the focusing of particles of different sizes. Taking advantage of the size-dependent particle lateral displacement, selective focusing of micrometer particles was demonstrated. This study systematically investigated continuous particle focusing in a groove-embedded microchannel. We expect that this device will be used for further applications, such as cell sensing and nanoparticle separation in biological and biomedical areas.

## 1. Introduction

Microfluidics is the science and technology of systems that studies fluid physics in channels with dimensions of tens to hundreds of micrometers [1,2]. Features such as small size [3], low reagent consumption [4], fast analysis [5], and low cost [6] make it suitable for various applications in different fields, such as drug delivery [7], point-of-care diagnosis [8], single cell virology [9], cell separation [10] and polymer synthesis [11]. Microfluidic particle focusing refers to the control over the positions of particles in a tight streamline, which can be divided into two-dimensional (2D) and three-dimensional (3D) focusing [12,13]. In 3D focusing, particles are focused both horizontally and vertically, two typical 2D cases [14]. Particle focusing techniques have gained increasing interest because particle focusing is an essential step for downstream processing, such as monitoring [15], imaging [16], counting [17] and separation [18]. Further, 3D hydrodynamic focusing microfluidics has been adopted for particle sensing applications such as cell detection [19]. In parallel with the development of particle focusing techniques, particle separation techniques have enabled various biological and life science applications [20,21,22,23,24]. For example, femtosecond pulse (fs) laser-based high-speed separation of fluorescence-activated human lung cancer A549 cells [25] and Raman image-activated separation of microalgal cells [26] based on intracellular metabolites have been achieved.

Microfluidic techniques for particle focusing or separation can be classified into two main groups depending on whether an external field is applied: active and passive methods [12,27]. Active techniques such as acoustophoresis [28], dielectrophoresis [29], magnetophoresis [30] and thermophoresis [31] use external forces to achieve particle focusing or separation. However, these techniques always need a complex fabrication process, and the external forces may induce negative effects, e.g., cell damage due to acoustic cavitation [32]. The passive methods such as deterministic lateral displacement (DLD) [33], inertial microfluidics [34], viscoelastic microfluidics [35] and hydrophoresis [36] rely on the channel structure or the fluidic properties to focus or separate particles. By now, it has been reported that DLD is able to separate exosomes or polystyrene particles down to 20 nm [37]. Size-based separation of hydrogel droplets [38], shape-based separation of microalgal cells [39] by inertial microfluidics, and shape-dependent separation of yeast *Saccharomyces cerevisiae* [40] by viscoelastic microfluidics have been achieved. Moreover, separation of blood cells [41] and the 9-µm polystyrene particles [42] by hydrophoresis in microchannels embedded with groove microstructures have also been reported.

There are several studies about particle focusing and separation using microchannels embedded with groove microstructures [43,44,45,46,47]. In 2009, Choi et al. successfully separated 4-µm polystyrene particles from 1-µm ones with hydrophoresis by focusing larger particles in a tight flow stream in an elastomeric microchannel with slanted grooves at the channel top [48]. Additionally, separation of 0.75- and 1.1-µm particles was demonstrated in a similar microchannel by ordering particles to two focused flow streams in a size-dependent manner [49]. Further, size-based separation of human leukemic HL 60 cells [50] and breast cancerous MCF-7 cells [51] were demonstrated in a channel with groove patterns. In recent years, herringbone groove structures have been increasingly employed for particle separation [52], detection [53] and mixing [54] in microfluidics. For instance, functionalization of a chip with herringbone grooves allows for specific and rapid separation of tumor-derived extracellular vesicles and analysis of their biological cargo [55]. More recently, we reported hydrodynamic focusing and separation of micrometer particles using fs laser-engraved open v-shaped microstructures [56]. Despite these recent advances, there is a lack of systematic study on how groove geometry affects particle lateral displacement, which is important to improve our understanding of the underlying focusing mechanisms and to guide the design and optimization of devices.

In this study, we engraved different types of groove microstructures on a glass substrate and studied their capabilities for focusing particles in microchannels (Re < 1). Atomic force microscopy (AFM) and scanning electron microscopy (SEM) were used to detect the groove structures and surface morphology. Both numerical simulation and experiments were conducted to investigate particle behaviors under the influence of groove geometry. The focusing performance proved to be influenced by particle size, groove angle and the middle open space between opposite grooves. This study systematically investigated the particle focusing in low Re-number flows, showing application potential in contact imaging, lifetime-resolved imaging, cell sensing and particle separation in biological and biomedical areas.

## 2. Materials and Methods

### 2.1. Design and Fabrication of Groove Microstructures

**Figure 1:**
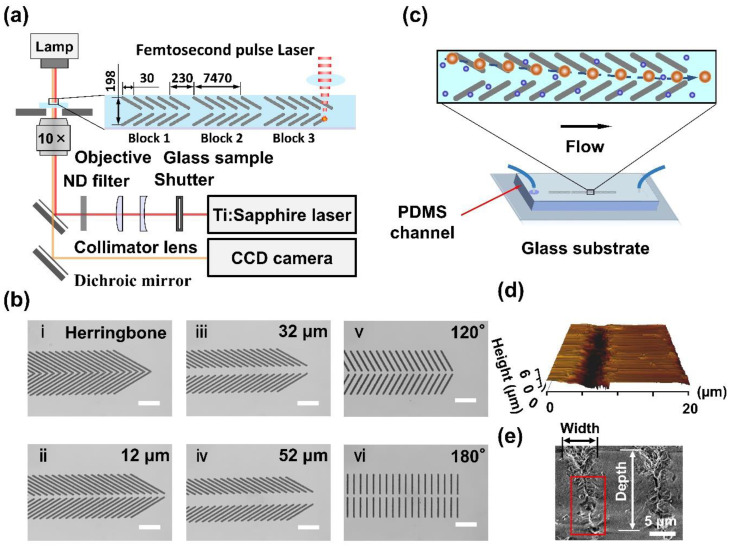
Femtosecond pulse laser engraved glass surface microstructures for selective particle focusing in a microchannel. (**a**) Schematic illustration of the laser fabrication system. (**b**) Six engraved microstructures of grooves: (**i**) herringbone, (**ii**–**iv**) 60° open v-shaped grooves with middle open spaces of ~12, 32 and 52 μm, and (**v**–**vi**) 120° and 180° open v-shaped with a middle open space of ~12 μm. Scale bar is 90 μm. (**c**) Schematic illustration of the microfluidic device used for selective particle focusing. (**d**) A 20 × 20-μm typical AFM image of the groove structure. (**e**) An SEM image of bird’s-eye view of the groove engraved by fs laser at 3.3 µJ/pulse.

Groove engraving of borosilicate glass was achieved by femtosecond (fs) laser ablation, as previously reported [56]. Laser pulses from a Ti:Sapphire fs laser amplifier (800 nm, 130 fs, 1 kHz, Solstice-Ref-MT5W, Spectra-Physics, Milpitas, CA, USA) were focused to a glass substrate (borosilicate, 76 × 26 × 1 mm, Matsunami Glass Ind., Ltd., Osaka, Japan) by an inverted microscope (IX71, Olympus, Tokyo, Japan) with a 10× objective lens (NA. = 0.25, Olympus). The diameter of the light irradiation spot was about 2.0 µm as a beam waist of laser, which was estimated from the numerical aperture of the objective. The fs micromachining of groove microstructures was conducted at 3.3 µJ/pulse. A motorized electric stage (Sigma Koki, E-65GR) was used at a speed of 100 µm/s, resulting in a pulse overlapping number of 20. Three blocks of the groove microstructures with a neighboring distance of 230 µm were fabricated on the surface of the glass substrate [see Figure 1a]. Each block consisted of 250 pairs of grooves with an interval distance of 30 µm, and a width of 198 µm. Six patterns of groove arrays with different structures were engraved: (i) herringbone grooves, (ii–iv) 60° open v-shaped grooves with 3 different middle open spaces of ~12, 32 and 52 μm, and (v–vi) open v-shaped grooves with 2 different angles, 120° and 180°; the middle open space was ~12 μm (Figure 1b).

### 2.2. Fabrication of Microfluidic Device

A 200-μm wide and 40-μm high polydimethylsiloxane (PDMS) microchannel was bonded to the groove-patterned glass substrate by plasma treatment (Plasma Cleaner CY-P2L-B), forming an enclosed microfluidic device (see Figure 1c). Before the bonding, the glass substrate was placed into tap water for 10-min ultrasonic cleaning to remove the surface debris that was produced due to the laser engraving. Then, the glass substrate was dried by nitrogen blast. Additionally, a control group was established using a glass substrate without groove patterns. The particle samples were injected into the channel via the inlet using a syringe pump (Harvard Apparatus 11 Elite). Particle positions across the channel width direction were recorded under an inverted microscope (Zeiss Axiovert 100 Microscope).

### 2.3. Particle Suspension Preparation

Polystyrene particles (diameter: 4.5 ± 0.3, 10 ± 1.0 and 15 ± 1.5 μm, density: 1.05 g/cm^3^, Polybead, Polysciences) and fluorescent polystyrene particles (diameter: 0.75 ± 0.02, 3.0 ± 0.15, 10 ± 1.0 μm, density; 1.05 g/cm^3^, Fluoreabright carboxylate YG, Polysciences) were diluted with water for all experiments. The particle suspensions were stored at 4 °C.

For evaluating the focusing performance of the device, 0.75-, 4.5-, 10- and 15-μm polystyrene particles were used. The original 4.5-, 10- and 15-μm particle solutions were diluted with pure water to ~5.0 × 10^5^ particles/mL. Unlike large particles, the free-falling velocity of 0.75-μm particles was slow for them to reach the channel bottom. Therefore, 0.75-μm particles could not be effectively influenced by localized secondary flows when flowing through the microchannel, causing them to be distributed at different height levels in the channel. To facilitate observation, the 0.75-μm original solutions were diluted to ~5.0 × 10^7^ particles/mL. The particles were injected into the channel at 500, 1000, 1500 and 2000 nL/min, respectively. Note that 15-μm particles were often clogged at 500 nL/min in the grooved microchannel, and the focusing experiment could not be performed at this flow rate.

To evaluate the influence of sedimentation rate on particle focusing performance, particle distributions at different height levels were recorded by the Photron high-speed camera at the end of block 3. The recording was performed at 3000 frames/second. As described before, 0.75-, 4.5-, 10- and 15-μm polystyrene particles were used. The particles were injected into the channel at the flow rates of 500 and 1000 nL/min, respectively. For 15-μm particles, the experiment was only performed at 1000 nL/min to avoid capture by the groove structures. The control groups without grooves in the channel were established for comparison. Additionally, 0.75-, 3.0- and 10-μm fluorescent particles were used for the sedimentation experiment in a 10-mL tube with approximate particle concentrations of 1.1 × 10^9^, 1.7 × 10^7^ and 5 × 10^5^ particles/mL.

To evaluate the ability of the device to selectively focus particles, 0.75-, 4.5-, 10- and 15-μm polystyrene particles were used. Mixtures of 0.75- and 10-μm particles were injected into the inlet at 500 nL/min. Mixtures of 4.5- and 15-μm were injected into the inlet at 1000 nL/min. The concentration of each type of particle was 2.5 × 10^5^ particles/mL. Particle distribution at the outlet was measured by the high-speed camera at 250 frames/s.

### 2.4. Cell Preparation and Viability Assays

Mouse myoblast cell line C2C12 (RCB0987) was used in our experiments. Cells were cultured using 5% CO_2_ at 37 °C with culture medium consisting of 10-mL Dulbecco’s Modified Eagle’s Medium (DMEM, 4.5 g/L glucose) with 10% fetal bovine serum (FBS) and antibiotic agents (100 units/mL penicillin, 100 μg/mL streptomycin). Cell diameter was calculated using ImageJ after detachment. The diameter was 15.3 ± 2.6 μm. Two hundred cells were measured for the calculation of diameter. The cells were freshly collected by trypsinization and resuspended in 5.0 × 10^5^ cells/mL medium and used within 3 h. Before the experiment, cell aggregates were removed by a 40-μm sterile strainer (EASYstrainer, Greiner Bio-One).

Cell viability assays were performed for C2C12 cells before and after flowing though the microchannel with groove arrays. Trypan blue solution with a concentration of 0.25% was used for quickly assessing cell membrane integrity-based viability in this study. Cell counting was performed with a hemocytometer, and the blue-dyed cells were regarded as dead. Viability was defined as the percentage of viable cells after and before the focusing experiment.

### 2.5. Groove Detection

To measure the groove morphology, atomic force microscopy (AFM, JPK instruments, NanoWizard 4) and scanning electron microscopy (SEM, Hitachi SU-1510) were employed. AFM detection was conducted under QI mode, using a pyramidal silicon tip (HQ-XSC11/No Al, MicroMasch). The radius, half-cone angle and height were 8 nm, 20° and 15 μm, respectively. Five grooves were detected after setting the scanning pixels at 256 × 256. The JPK data processing 6.1 software was used for calibration of the scanned data. It showed that the groove was 5.6 ± 0.4 μm in width and 3.8 ± 0.7 μm in depth at the engraving energy of 3.3 μJ/pulse (Figure 1d). SEM detection was conducted at 1.0 kV accelerating voltage, and the data were shown as mean ± standard deviation (SD) of 5 measurements. SEM results showed that the groove was 6.4 ± 0.2 μm in width and 16.7 ± 0.8 μm in depth (Figure 1e). The SEM visualization demonstrated good agreement with a previous report [56]. A slit observed within the red box (Figure 1e) was due to the laser self-focusing effect. A significant difference was found in the groove depth detected by AFM and SEM because AFM detection was limited by the probe height limitation of 15 μm and the tip half-cone angle of 20°.

## 3. Results

### 3.1. Numerical Simulation of Fluidic Behaviors

Numerical simulation was conducted to investigate the effects of groove angle and the middle open space on non-buoyant particle focusing behaviors. The rigid polystyrene particles with a mass density of 1.05 g/cm^3^ were used in the focusing experiment. Simplified 3D models were established with the software COMSOL Multiphysics 5.4 based on the groove dimensional data in SEM images, which simulated fluid motion dynamics caused by the groove structures. Additionally, we built a control model consisting of a plain microchannel without patterned grooves. The injection flow rate was set at 1000 nL/min using water as a Newtonian fluid.

**Figure 2:**
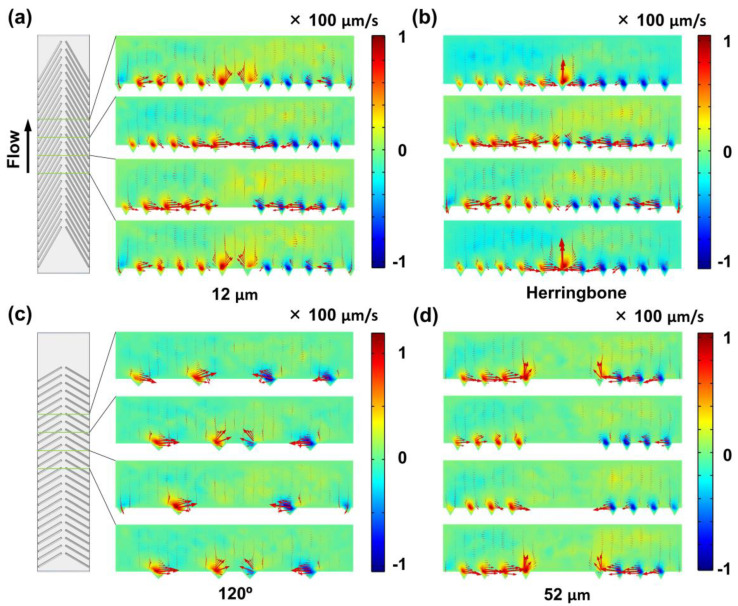
Numerical simulation of 2D vector plots of flow velocity fields at cross-sections of the microchannels with fs laser-engraved grooves. (**a**) 60° open v-shaped grooves with the middle open space of ~12 μm. (**b**) 60° herringbone grooves. (**c**) 120° open v-shaped grooves with the middle open space of ~12 μm. (**d**) 60° open v-shaped grooves with the middle open space of ~52 μm. The red arrows show the proportional velocity and the direction of its vector.

Two-dimensional vector plots of flow velocity fields at cross-sections of the microchannels were visualized. Similar localized secondary flows induced by grooves were found in the 12-μm and herringbone groups, which tended to direct the motion of the non-buoyant particles locating close to the channel bottom toward the center of the microchannel (Figure 2a,b). Compared to the 120° group, there were more grooves in the 12-μm group, giving it stronger capability to drive particles in the lateral direction (Figure 2a,c). Almost no secondary flows were found in the 180° and control groups (Figure S1b,c). The secondary flows that directed fluid motion toward the center were mainly at the channel bottom close to the grooves, which were dependent on the middle opening (Figure 2a,d and Figure S1a). Interestingly, strong upward flows were revealed in the herringbone group compared to the 60° group (Figure 2a,b and Figure S1d). Because there were upward fluid flows in the channel center, the particles flowing above were expected to be unstable due to fluid-induced hydrodynamic forces. Therefore, we used groove arrays with middle openings in the experiment. These simulation results revealed that the secondary flows were effective at the channel bottom and dependent on the middle open space and the groove angle. The simulation was of significance in the study because it investigated the effects of groove geometries (e.g., angle and middle open space) on the localized secondary flows, which provided the main driving forces for particle lateral displacement.

### 3.2. Effect of the Middle Open Space

**Figure 3:**
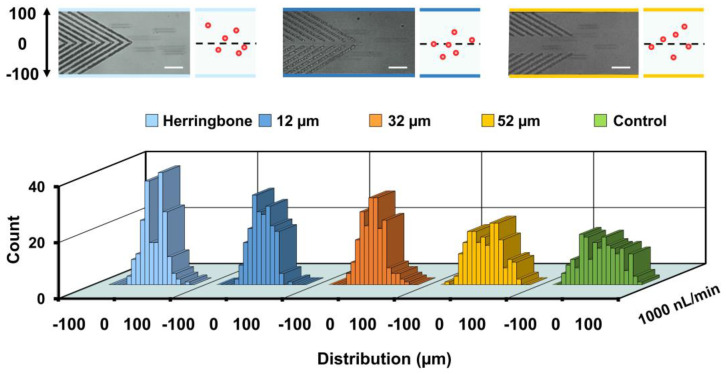
Particle focusing performance influenced by the middle open space between the opposite grooves. Distributions of 15-μm polystyrene particles at the end of block 1 of groove arrays having four different middle open spaces: 0 (herringbone), ~12, 32, and 52 µm, when the injection flow rate is 1000 nL/min. The control refers to a microchannel without groove microstructures. Superimposed experimental images and schematics for the herringbone, 12-, 52-µm grooves are shown at top. Scale bar is 60 µm. Two hundred particles are measured for each group.

The effect of the middle open space between two opposite grooves on particle lateral displacement was explored. Fifteen-micrometer polystyrene particles were injected into the channel and observed at the end of block 1 at 1000 nL/min (Re = 0.15). The results showed that the distribution of particle lateral positions was different (Figure 3): control (0.2 ± 45.4 µm), 52-µm (−0.4 ± 35.5 µm), 32-µm (−2.7 ± 26.5), 12-µm (0.1 ± 23.6 µm) and herringbone (0-µm, −0.6 ± 25.2 µm). The herringbone grooves and grooves with 12-µm open space showed better focusing ability, while strong two-peak distributions were found for the herringbone group. Moreover, an accumulative effect of groove number on particle focusing was found in a microchannel having three consecutive blocks (see Figure S2a), which agreed with the findings in our previous work [56]. Polystyrene particles randomly distributed at the channel inlet were able to be gradually directed towards the channel center at the outlet (see Figure S2). Interestingly, double-peak distributions were found at the end of block 1, block 2 and the outlet.

### 3.3. Effect of Groove Angle

**Figure 4:**
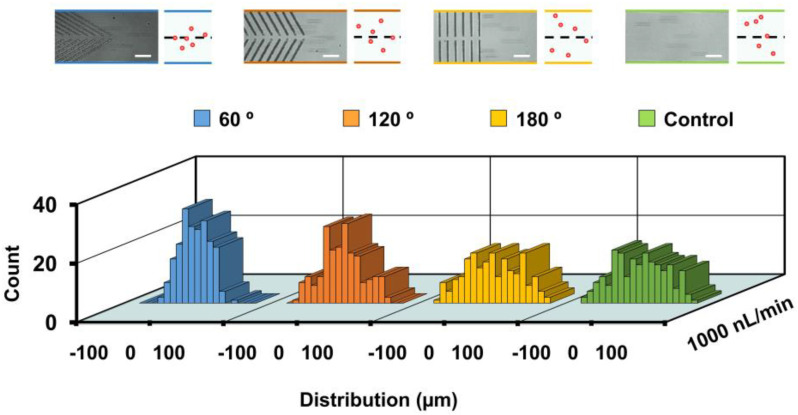
Particle focusing performance affected by groove angle. Distributions of 15-μm polystyrene particles at the end of block 1. Superimposed experimental images and schematics are shown at the top. Two hundred particles are measured for each group. Scale bar is 60 μm.

To investigate the effect of the groove angle on particle focusing behaviors, groove arrays of different angles, 60°, 120°, 180° and control (a channel without microstructures) were used. Polystyrene particles of 15 um in diameter were injected into the channel at 1000 nL/min. Particle distributions at the end of block 1 were analyzed and compared. It showed that the focusing performance was increased with decreases in groove angle (Figure 4). Particles were focused to a narrower stream in the 60° group (0.1 ± 23.6 µm) than that in the 120° group (−3.0 ± 36.7 µm), whereas particles were randomly distributed at the end of block 1 for the control (0.2 ± 45.4 µm) and 180° (0.9 ± 46.3 µm) groups. We noted that the focusing performance of the groove-embedded microchannel decreased with increases of flow rate (see Figure S3), suggesting that the microchannel was more suitable for particle focusing at relatively low flow rates. The higher flow rate of the fluids along the channel was expected to enable a higher particle flowing rate, reducing the time period of interaction between particles and groove-induced secondary flows. Moreover, the particles had less time to settle to the channel bottom, where the secondary flow was more effective when flowing through the microchannel at higher speed. This may have accounted for the better focusing performance at a relatively lower injection flow rate.

**Figure 5:**
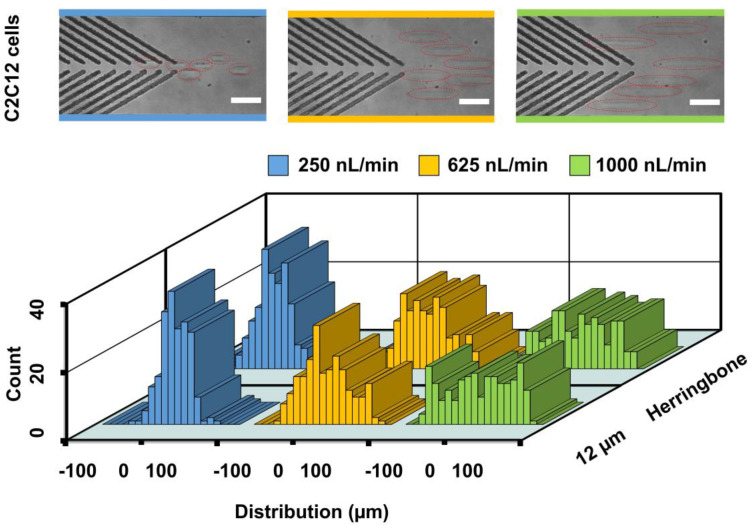
Distributions of C2C12 cells at the end of block 1 in the channel. The injection flow rates were 250, 625 and 1000 nL/min, respectively. The herringbone and 12-µm microstructures were used. Superimposed experimental images are shown for the cell focusing by 12-um group. The red dotted ellipses denote cells. Two hundred cells were analyzed for each group. Scale bar is 60 µm.

Besides polystyrene particles, this microchannel with embedded groove arrays was able to focus living cells. C2C12 cells were injected into microchannels with grooves of either herringbone or the 12-μm open space. Results showed that C2C12 cells were able to be driven closer toward the channel center for the herringbone (−2.6 ± 24.1 µm) and 12-µm (0.4 ± 21.7 µm) groups when the flow rate was 250 nL/min (Figure 5). The distributions of cell lateral positions at 625 nL/min (−0.4 ± 36.8 µm) and 1000 nL/min (0 ± 46.2 µm) became increasingly wider in the herringbone groups. The focusing of 15-μm polystyrene particles at 1000 nL/min was better than that of the cells. The intrinsic cell properties, such as shape, stiffness and mass density, may have accounted for the difference in the focusing performance. Moreover, a lower flow rate enabled the cells to interact with the secondary flow induced by grooves for a relatively longer time period, which may have accounted for the better focusing performance at lower flow rates (e.g., 250 nL/min). A similar tendency was found at 625 nL/min (−0.2 ± 35.4 µm) and 1000 nL/min (1.7 ± 47.7 µm) in the 12-µm groups. Additionally, we found that this device did not have a significant effect on cell viability (93.2%).

### 3.4. Effect of Particle Size

To explore the capability of the device in focusing particles of different sizes, 15-, 10, 4.5- and 0.75-μm polystyrene particles were injected into the channel with 60° groove arrays and the channel without groove microstructures (control) at 1000 nL/min. The distance between the two opposite grooves was ~12 μm. Particle distributions were analyzed at the end of block 3 (outlet). Additionally, particle distributions at different height layers were recorded.

**Figure 6:**
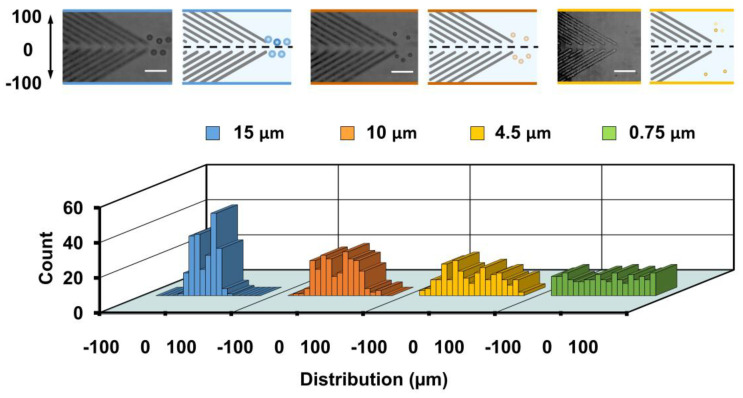
Particle distributions at the end of block 3 (outlet) with an injection flow rate of 1000 nL/min. Histograms of the lateral distributions of 15-, 10-, and 4.5- and 0.75-μm polystyrene particles are shown. Two hundred particles are measured for each group. Superimposed experimental images and schematics of particles at the layer 15 μm above the bottom base in the channel with groove structures are presented. Scale bar is 60 μm.

The focusing performance was found to decrease with decreases in particle size (Figure 6, bottom). Two-peak distributions were found when 10-μm particles were used (0.1 ± 33.0 μm). Distributions of 4.5-μm (0 ± 48.9 μm) and 0.75-μm (0.3 ± 60.0 μm) particles became increasingly wider. Further, the focusing performance was able to be enhanced by decreasing the flow rate to 500 nL/min (see Figure S4). However, more and more 15-μm particles were trapped by the groove confinement when we further reduced the flow rate. No focusing performance was found for 0.75-μm particles when the injection flow rate was 500 nL/min (see Figure S4).

On the other hand, it was shown that the 15- and 10-μm polystyrene particles were not focused in the control groups at 15 μm above the bottom base layer (Figure S5a). An in-focus 15-um particle close to the channel center was observed in the channel with groove structures. In-focus 4.5-um particles could be observed in both channels with groove structures (Figure 6, top) and without groove structures (Figure S5a) when the injection flow rate was 1000 nL/min. This result showed that relatively small particles sometimes cannot reach the bottom of the microchannel where the secondary flows are most effective. A video demonstrating the in-focus and out-of-focus 4.5-μm particles at 1000 nL/min was recorded at a layer 15 μm above the bottom base layer (Video S1). A similar phenomenon was found when the injection flow rate was set at 500 nL/min (Figure S5b). Additionally, distributions of 0.75-μm particles were visualized at different height layers along the channel height direction. We observed that 0.75-μm particles could locate in all layers (bottom base layer, 23 and 33 μm above the bottom base layer) in the channels with or without groove microstructures. Videos recording the 0.75-μm particles flowing 33 μm above the bottom base layer in the channel with groove microstructures are provided, in which the injection flow rates were 1000 and 500 nL/min (Videos S2 and S3). The software ImageJ was used to brighten and improve the contrast of the movies. Additionally, a sedimentation experiment was performed using 0.75-, 3.0- and 10-μm fluorescent particles. It showed that the downward sedimentation rate decreased significantly with decreases in particle size (Figure S6). Size-dependent lateral displacement was found in this study. Larger particles such as the 10- and 15-μm ones needed less time to reach the channel bottom and thus were subjected to groove microstructure-induced secondary flows that directed particles toward the channel center.

Free-falling velocity of a rigid-sphere particle in the gravitational direction (*V_y_*) can be determined by Stokes’ Law in Newtonian fluids at low Reynolds numbers [57,58]: *V_y_* = 2*g(ρ_s_-ρ)r*^2^/9*η*, where *g* is the gravitational constant, *ρ_s_* is the particle density, *ρ* is the fluid density, *r* is the particle radius and *η* is the fluid dynamic viscosity. The sedimentation rate is proportional to the square of the particle radius, suggesting that larger particles need less time to reach the channel bottom [59]. On the other hand, the fluid motion at the bottom layer was arranged toward the channel center by the groove microstructures. Thus, larger particles such as the 10- and 15-μm ones locating closer to the bottom of the microchannel were more likely to be driven toward the channel center by the hydrodynamic drag forces defined as *F_d_* = 6*πηrv*, where *v* is the relative velocity between particles and fluids [60].

### 3.5. Selective Particle Focusing

**Figure 7:**
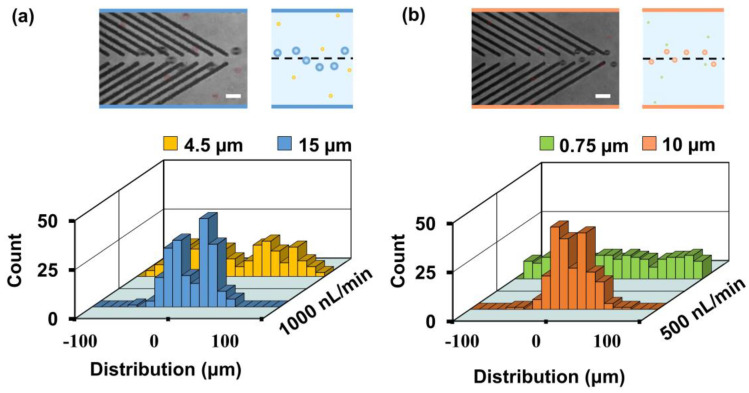
Selective particle focusing enabled by open v-shaped microstructures. (**a**) Distributions of 4.5- and 15-μm polystyrene particles at the end of the third block (outlet). The injection flow rate is 1000 nL/min. (**b**) Distributions of 0.75- and 10-μm polystyrene particles at the outlet. The injection flow rate is 500 nL/min. Red circles denote the positions of the 4.5- and 0.75-μm particles. Two hundred particles are measured for each group. Superimposed experimental images and schematics are shown. Scale bar is 30 μm.

Based on the size-dependent focusing performance, selective particle focusing was achieved using the 12-μm open v-shaped groove microstructures. Results showed that the distribution of the 15-μm polystyrene particles (0 ± 22.5 μm) was close to the channel center, whereas the 4.5-μm particles were almost randomly distributed (−0.5 ± 50.5 μm) at the outlet when the injection flow rate was 1000 nL/min (Figure 7a). Moreover, focusing of 10-μm particles was demonstrated at an injection flow rate of 500 nL/min (Figure 7b). This showed that the 0.75-μm particles were randomly distributed (1.5 ± 56.1 μm), while the 10-μm particles were directed to a focal stream (2.2 ± 20.2 μm) close to the channel center (see Video S4).

## 4. Discussion

Numerical simulations revealed that the secondary flows were stronger in the area close to the groove microstructures at the channel bottom. When the middle open space between the opposite grooves was wider, e.g., under 32- and 52-μm conditions, the center-driven tendency of the fluids became weaker, resulting in reduced particle focusing performance. However, with a decrease in the width of the middle space between the opposite grooves, e.g., in 12-μm and herringbone conditions, the upward flows in the channel center became stronger. For a 15-μm polystyrene particle, the free-falling velocity or terminal rate *V_y_* was about 6.6 μm/s, but the velocity of the upward fluids could reach over 80 μm/s (estimated by numerical simulation in the herringbone condition), which was expected to make particles located above the channel center area unstable [61]. As a result, significant two-peak distributions were observed in the channel with herringbone grooves.

Size-dependent lateral displacement was found in this study. Larger particles such as the 10- and 15-μm ones needed less time to reach the channel bottom, thus becoming subject to groove microstructure-induced secondary flows that directed particles toward the channel center. However, more time was required for the smaller particles. For example, the free-falling velocity of 0.75-μm polystyrene in water under current experimental conditions was~16 nm/s. This resulted in particles locating at different height levels, unfocused in the channel. Differences in the distribution of 10- and 15-μm particles were found, which may have been due to differences in their ability to interact with the upward fluid flows in the channel center area. Based on the size-dependent lateral displacement, focusing of micrometer particles was demonstrated. To achieve further particle separation, purity will need to be improved because both large and small particles appeared at the area close to the channel center. We anticipate this device can be used for nano-sized bacteria separation based on the previously developed acoustic focusing system [62] or hydrodynamic focusing technique [63].

## 5. Conclusions

In this study, we investigated particle focusing at low Re conditions in microchannels with fs laser-engraved glass groove microstructures. Experimental results showed that the focusing performance is influenced by the groove number, the middle open space between opposite grooves, groove angle and particle size. We expect that this device can be used for applications where particle and cell focusing at relatively low flow-rate conditions are required, such as contact imaging and lifetime-resolved imaging. Micrometer-scale particles require less time to reach the channel bottom to interact with localized center-driven secondary flows, whereas nanometer-scale particles need relatively longer time to reach the channel bottom due to their lower free-falling velocity, resulting in variation in the vertical positions of the particles in the channel height direction. Selective focusing of micrometer-scale polystyrene particles was demonstrated. Further optimization of the device in terms of groove angle and channel geometry will be required for improved focusing performance. This study systematically investigated the particle focusing in low Re-number flows in groove-embedded microchannels, which are expected to be used for future applications such as cell sensing and nanoparticle separation in the biological and biomedical fields.

## Data Availability

The data presented in this study are available on request from the corresponding author.

## Supplementary Materials

The following are available online at https://www.mdpi.com/article/10.3390/bios11080263/s1, Figure S1: Numerical simulation of 2D vector plots of flow velocity fields at cross-sections of the microchannels with fs laser engraved grooves having different geometries. (a) 60° open v-shaped grooves with the middle open space of ~32 μm. (b) 180° open v-shaped grooves with the middle open space of ~12 μm. (c) Control group without groove structures. The red arrows show the proportional velocity and the direction of its vector. (d) Comparison of upward flow rates for a center point at five micrometers above the channel bottom for the herringbone and 12-μm groups. 25 points were used for the calculation. The injection flow rate is 1000 nL/min; Figure S2: Particle focusing performance enhanced by the groove number. (a) Schematic illustration of a microfluidic channel with three blocks of groove microstructures. Superimposed experimental images and schematics are shown. Scale bar is 60 µm. 12 μm denotes the distance of the middle open space between the opposite groove. (b) Plots of the lateral distributions of particles along the channel width. The distributions of particles at four different locations are recorded and compared: inlet (−1.5 ± 48.1 µm), after passing block 1 (−1 ± 26.0 µm), block 2 (1.2 ± 23.1 µm), and block 3 (outlet, −1 ± 20.2 µm). 200 particles are measured for each group; Figure S3: The effect of groove angles on particle distributions at the end of block 1 when the injection flow rates are 1500 and 2000 nL/min. 200 particles were analyzed for each group; Figure S4: Plots of the lateral distributions of polystyrene particles having different sizes:10-µm (2.5 ± 26.7 µm), 4.5-µm (0.4 ± 41.9 µm) and 0.75-µm (−0.2 ± 59.9 µm) in a microchannel with fs laser engraved 60° grooves. The injection flow rate is 500 nL/min. Particle lateral positions were detected at the end of block 3 (outlet). 200 particles are measured for each group; Figure S5: Particle distributions at different height levels at the end of block 3 (outlet). (a) Distributions of 15-, 10- and 4.5-μm polystyrene particles in the channels without groove microstructures. The focal position is 15 μm above channel bottom. The injection flow rate is 1000 nL/min. (b) Distributions of different sized polystyrene particles at different height levels: bottom (upper pictures) and 15 μm above channel bottom base (lower pictures), when the injection flow rate is 500 nL/min. (i,ii) Distributions of (i) 10- and (ii) 4.5-μm polystyrene particles in the channel with groove structures. (iii–v) Distributions of (iii) 15-, (iv) 10- and (v) 4.5-μm in the channels without groove structures. Bottom base refers to the position where a particle is in focus under a static condition. 15- and 10-μm particles are observed under a 40 × objective lens and 4.5-μm particles are observed under a 60× objective lens. Scale bar is 60 μm; Figure S6: Particle sedimentation in 10-mL tubes at different time points. Particle concentrations are 5 × 105, 1.7 × 107, 1.1 × 109 particles/mL for 10.0-, 3.0- and 0.75-μm fluorescent particle solutions, respectively. Video S1: 4.5-μm particles flowing at 1000 nL/min; Video S2: 0.75-μm particles flowing at 1000 nL/min; Video S3: 0.75-μm particles flowing at 500 nL/min; Video S4: selective focusing of 10-μm particles.
