# Supplementary material for: Focusing of Particles in a Microchannel with Laser Engraved Groove Arrays
Source: Biosensors (Basel). 2021 Aug 4;11(8):263. doi: 10.3390/bios11080263 (PMC8392838; doi:10.3390/bios11080263)
Supplement: Supplementary file 1 [file biosensors-11-00263-s001.zip › biosensors-1309326-supplementary.pdf]

Focusing and separation of particles in a microchannel with laser engraved arrays

Tianlong Zhang[1,2], Yigang Shen[3], Ryota Kiya[1], Dian Anggraini[1], Tao Tang[1], Hanaka Uno[1], Kazunori Okano[1], Yo Tanaka[3], Yoichiroh Hosokawa[1], Ming Li[2*], and Yaxiaer Yalikun[1*]

1. Division of materials science, Graduate School of Science and Technology, Nara Institute of Science and Technology, 630-0192, Ikoma, Japan
2. School of Engineering, Macquarie University, Sydney 2122, Australia
3. Center for Biosystems Dynamics Research, RIKEN, Osaka 565-0871, Japan

*Email: ming.li@mq.edu.au
yaxiaer@ms.naist.jp

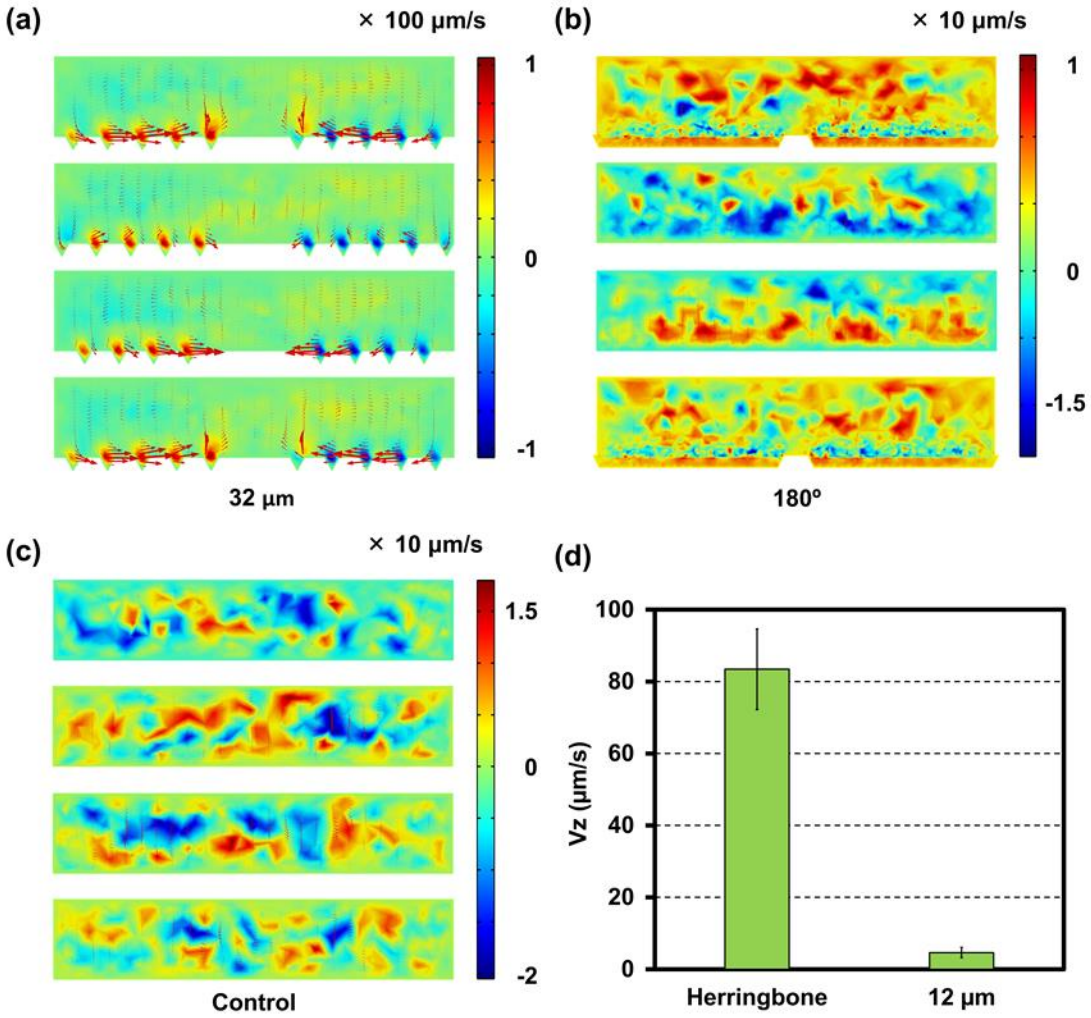

Figure S1 Numerical simulation of 2D vector plots of flow velocity fields at cross-sections of the microchannels with fs laser engraved grooves having different geometries. (a) 60º open v-shaped grooves with the middle open space of ~ 32 μm. (b) 180º open v-shaped grooves with the middle open space of ~ 12 μm. (c) Control group without groove structures. The red arrows show the proportional velocity and the direction of its vector. (d) Comparison of upward flow rates for a center point at five micrometers above the channel bottom for the herringbone and 12-μm groups. 25 points were used for the calculation. The injection flow rate is 1000 nL/min.

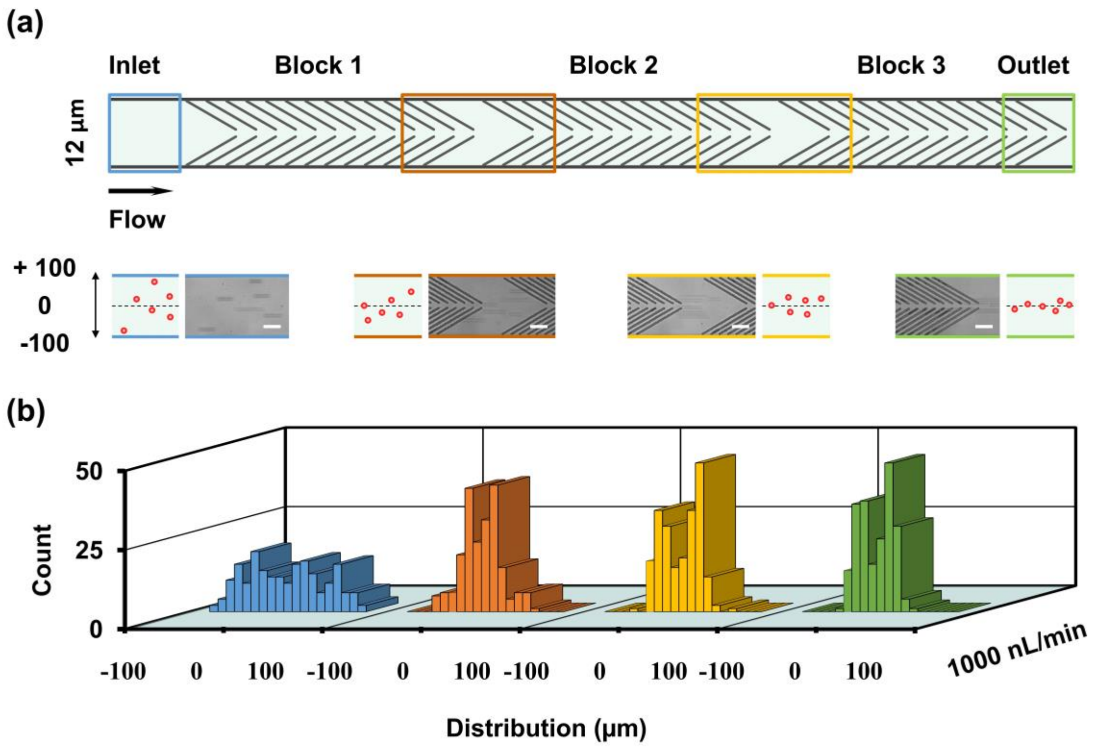

Figure S2 Particle focusing performance enhanced by the groove number. (a) Schematic illustration of a microfluidic channel with three blocks of groove microstructures. Superimposed experimental images and schematics are shown. Scale bar is 60 µm. 12 µm denotes the distance of the middle open space between the opposite groove. (b) Plots of the lateral distributions of particles along the channel width. The distributions of particles at four different locations are recorded and compared: inlet (-1.5 ± 48.1 µm), after passing block 1 (-1 ± 26.0 µm), block 2 (1.2 ± 23.1 µm), and block 3 (outlet, -1 ± 20.2 µm). 200 particles are measured for each group.

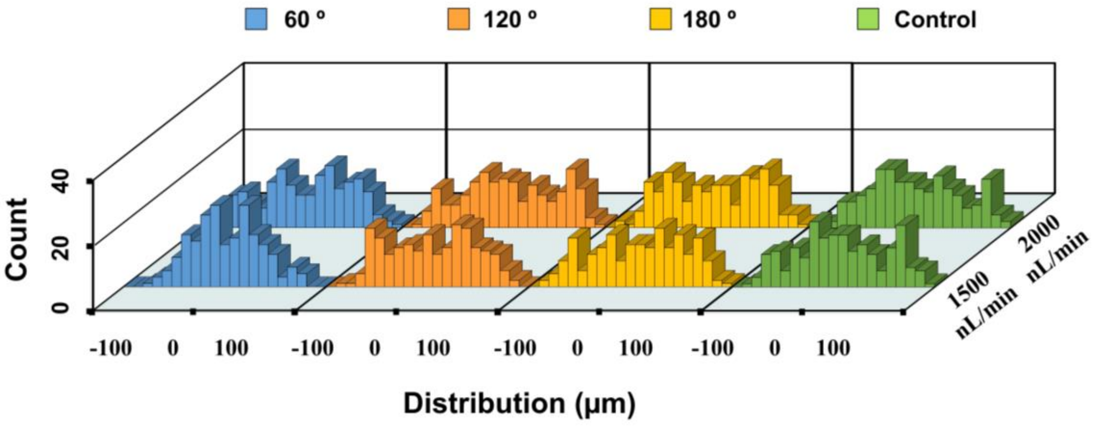

Figure S3 The effect of groove angles on particle distributions at the end of block 1 when the injection flow rates are 1500 and 2000 nL/min. 200 particles were analyzed for each group.

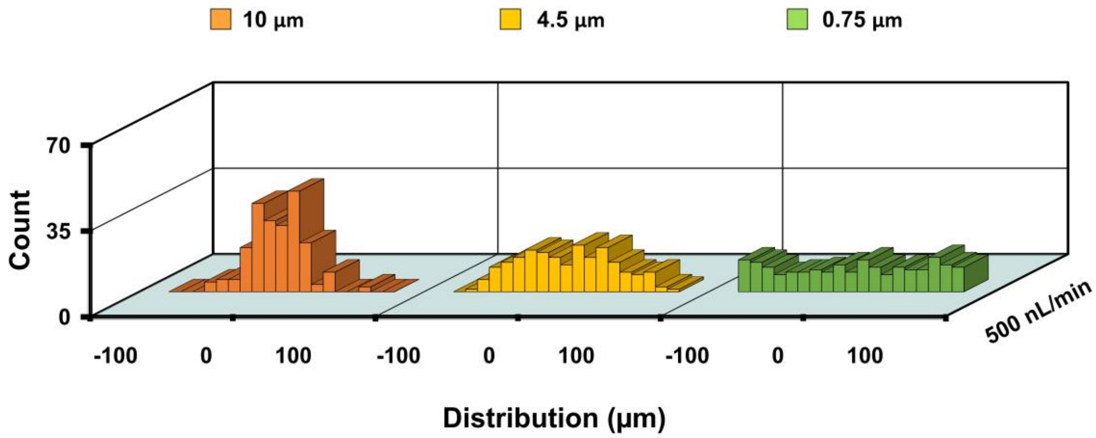

Figure S4 Plots of the lateral distributions of polystyrene particles having different sizes: 10-µm (2.5 ± 26.7 µm), 4.5-µm (0.4 ±41.9 µm) and 0.75-µm (-0.2 ± 59.9 µm) in a microchannel with fs laser engraved 60° grooves. The injection flow rate is 500 nL/min. Particle lateral positions were detected at the end of block 3 (outlet). 200 particles are measured for each group.

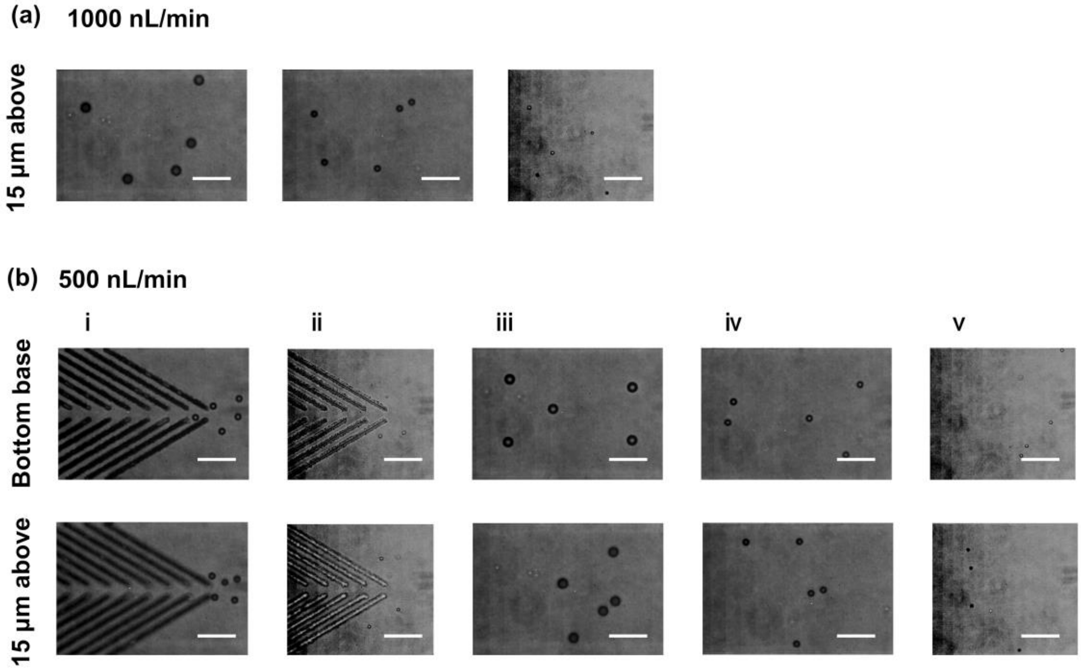

Figure S5 Particle distributions at different height levels at the end of block 3 (outlet). (a) Distributions of 15-, 10- and 4.5-µm polystyrene particles in the channels without groove microstructures. The focal position is 15 µm above channel bottom. The injection flow rate is 1000 nL/min. (b) Distributions of different sized polystyrene particles at different height levels: bottom (upper pictures) and 15 µm above channel bottom base (lower pictures), when the injection flow rate is 500 nL/min. (i, ii) Distributions of (i)10- and (ii) 4.5-µm polystyrene particles in the channel with groove structures. (iii-v) Distributions of (iii) 15-, (iv)10- and (v) 4.5-µm in the channels without groove structures. Bottom base refers to the position where a particle is in focus under a static condition. 15- and 10-µm particles are observed under a 40 × objective lens and 4.5-µm particles are observed under a 60× objective lens. Scale bar is 60 µm.

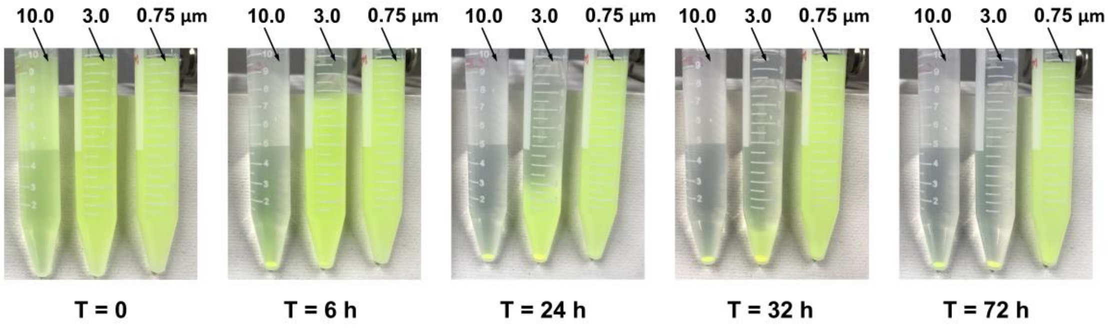

Figure S6 Particle sedimentation in 10-mL tubes at different time points. Particle concentrations are 5×10^5, 1.7×10^7, 1.1×10^9 particles/mL for 10.0-, 3.0- and 0.75-μm fluorescent particle solutions, respectively.
